# Involvement of GABAergic and Serotonergic Systems in the Antinociceptive Effect of Jegosaponin A Isolated from *Styrax japonicus*

**DOI:** 10.3390/molecules28052243

**Published:** 2023-02-28

**Authors:** Lei He, Ying Zhou, Li Ma, Wencui Wang, Lei Yao

**Affiliations:** 1 Department of Resources and Environment, School of Agriculture and Biology, Shanghai Jiao Tong University, 800 Dong Chuan Road, Shanghai 200240, China; 2 Department of Landscape Architecture, School of Design, Shanghai Jiao Tong University, 800 Dong Chuan Road, Shanghai 200240, China

**Keywords:** *Styrax japonicus*, jegosaponin A, antinociceptive, GABAergic, serotonergic

## Abstract

The antinociceptive activity of the flower extracts of *Styrax japonicus* was confirmed in our previous study. However, the key compound for analgesia has not been distinguished, and the corresponding mechanism is obscure. In this study, the active compound was isolated from the flower by multiple chromatographic techniques and structurally illustrated using spectroscopic methods and referring to the related literature. The antinociceptive activity of the compound and the underlying mechanisms were investigated using animal tests. The active compound was determined to be jegosaponin A (JA), which showed significant antinociceptive responses. JA was also shown to possess sedative and anxiolytic activities but no anti-inflammatory effect, implying the association of the antinociceptive effects with the sedative and anxiolytic activities. Further antagonists and calcium ionophore tests showed that the antinociceptive effect of JA was blocked by flumazenil (FM, antagonist for GABA-A receptor) and reversed by WAY100635 (WAY, antagonist for 5-HT_1A_ receptor). Contents of 5-HT and its metabolite (5-HIAA) increased significantly in the hippocampus and striatum tissues after JA administration. The results indicated that the antinociceptive effect of JA was regulated by the neurotransmitter system, especially GABAergic and serotonergic systems.

## 1. Introduction

Extracts from leaves and stems of *Styrax japonicus* Siebold & Zucc. (Styracaceae) have been reported to contain various active compounds of medicinal values. Among them, 2-hexenal, n-hexaldehyde and nerol, for instance, could strongly inhibit bacteria growth [1]. Lignan styrlignan A and lignan glycosides styraxjaponoside A and B inhibited the activity of HIV-1, especially styraxjaponoside B, which effectively blocked the fusion of HIV-1 with immune cells [2,3]. A norlignane and styraxlignolide A displayed significantly anticomplement activities [4], and terpenoid styraxoside A showed anti-inflammatory activities [5]. Nevertheless, the medical value of compounds from flowers is seldom explored. *Styrax japonicus* flowers have been used as Chinese folk medicine to treat pain as recorded in the Compilation of National Chinese Herbal Medicine [6]. In our previous study, the ethanol extract of *Styrax japonicus* flowers was confirmed to display strongly antinociceptive effects in mouse writhing and formalin tests [7]. However, the key compound for analgesia has not been distinguished and the corresponding mechanism is not completely uncovered.

According to the definition of neurophysiology, pain is a kind of sensory information of the body tissue damage [8]. In the conduction of pain signals, the information is always reflected in brain tissues, including the hypothalamus, hippocampus, striatum and cerebral cortex, which receive or generate signal molecules (dopamine, adrenaline, GABA or 5-HT) to regulate the perception of pain [9,10]. It is known that natural products exert antinociceptive effects on mice with various systems involved, such as the opioid system, ion channels and neurotransmitter systems [11,12,13,14] and with other effects intertwined, such as anti-inflammatory, sedative and anxiolytic activities [15,16,17]. Therefore, the underlying mechanisms of analgesia of compounds from *Styrax japonicus* flowers deserve in-depth investigation.

In this study, the glycosides derived from the ethanol extracts of flowers were further fractionated. The target compound was enriched employing chromatography coupled with animal model assays for screening its antinociceptive effect. The molecular structure was determined by ultrahigh-performance liquid chromatography Q extractive mass spectrometry (UHPLC-QE-MS), infrared (IR), nuclear magnetic resonance (NMR) and by referring to the related literature. The pentobarbital sodium-induced sleep test and the mouse hind paw edema test were carried out to evaluate the sedative activity and anti-inflammatory effect of the target compound. A23187, the calcium ionophore; glibenclamide (GBM), the ATP-sensitive K^+^ channel inhibitor; capsazepine (CZP), the antagonist of transient receptor potential vanilloid type1 (TRPV1); naloxone hydrochloride (NXH), the non-selective antagonist of opioid receptors; UFP-101 (UFP), the selective antagonist of the NOP receptor; FM, the antagonist of the GABA-A receptor; SCH23390 (SCH), the antagonist of the dopamine D1 receptor; and WAY, the antagonist of the 5-HT_1A_ receptor were used to investigate the possible mechanism involving calcium ion influx, ATP-sensitive K^+^ channels, TRPV1, opioid system, as well as GABAergic, dopaminergic and serotoninergic systems. Then, the contents of relevant neurotransmitters were quantified in different brain tissues of mice. In addition, open field and elevated plus maze tests were applied to evaluate the anxiolytic response of the active compound.

## 2. Results

### 2.1. Screening of Antinociceptive Components

**Figure 1:**
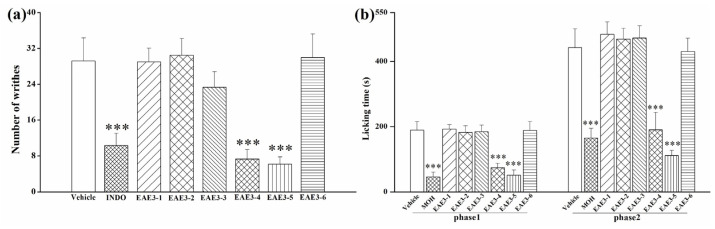
Antinociceptive effects of fractions of EAE3 in antinociceptive tests. 10 mg/kg of fractions from EAE3 in the writhing test (**a**) and the formalin test (**b**). Vehicle: blank control, INDO: indomethacin, MOH: morphine hydrochloride. Data are displayed as mean ± SD, *** *p* < 0.001 vs. vehicle group.

Antinociceptive effects of the sub-extracts, petroleum ether extract (PE), ethyl acetate extract (EAE), n-butanol extract (NBE) and aqueous residues (AR) from the ethanol extract are shown in Figure S1a,b. EAE and NBE (glycosides as their main components) reduced the number of writhes (*p* < 0.001) in the writhing test and the licking time in both phases of the formalin test significantly (*p* < 0.001). The higher yielding EAE was further separated by the D101 macroporous resin, and the fraction EAE3 displayed extremely strong antinociceptive activities in both animal models (Figure S1c,d). After fractionation by the silica gel column chromatography, six fractions of EAE3 were obtained. Considerable antinociceptive responses were observed in groups treated with EAE3-4 and EAE3-5 in animal tests (Figure 1a,b). Then, EAE3-5 was repeatedly separated by semi-preparative HPLC and mainly two obvious peaks appeared in the chromatograph, which were supposed to represent two compounds (i.e., Compound **1** and Compound **2**). After animal pre-tests, Compound **1** was tentatively determined to be the antinociceptive component from the flower extract.

### 2.2. Identification of Compound ***1***

**Figure 2:**
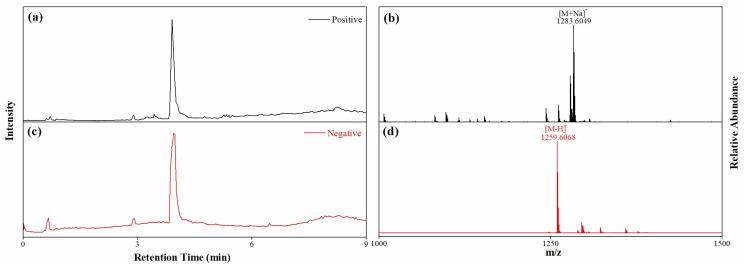
Chromatograms and exact masses of Compound **1** in positive (**a**,**b**) and negative (**c**,**d**) modes.

**Figure 3:**
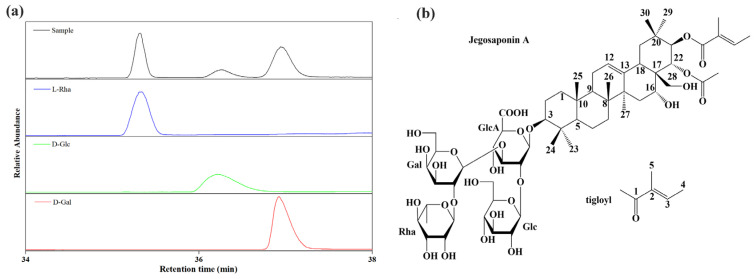
GC-MS chromatograms of sugar groups (**a**) and molecular structure of jegosaponin A (**b**). L-Rha: L-rhamnose, D-Glc: D-glucose, D-Gal: D-galactose.

Figure 2 displays the chromatograms from UHPLC-QE-MS in two ion modes. The molecular formula of Compound **1** was determined as C_61_H_96_O_27_ based on exact masses of [M+Na]^+^ at *m*/*z* 1283.6049 and [M−H]^−^ at *m*/*z* 1259.6068. Additionally, the molecule might contain two hydroxyl, one methylpentose, two hexose and one hexuronic acid groups according to fragment ions (Figure S2). GC chromatograms of the acid-hydrolyzed samples matched well with that of the standards (Figure 3), indicating the presence of L-rhamnopyranosyl, D-glucopyranosyl and D-galactopyranosyl groups in Compound **1**, and a glucuronic acid moiety was conformed in sugar units by NMR spectrums (Figures S3–S6). In addition, the information (Figure S7) from IR revealed that the molecule has multiple carbonyl moieties. Finally, Compound **1** was identified as JA according to ^1^H-NMR and ^13^C-NMR spectral data (Table 1) and the 2D NMR information (Figures S4–S6) as well as previous literature [18,19].

### 2.3. Effects of Jegosaponin A in Antinociceptive Tests

**Figure 4:**
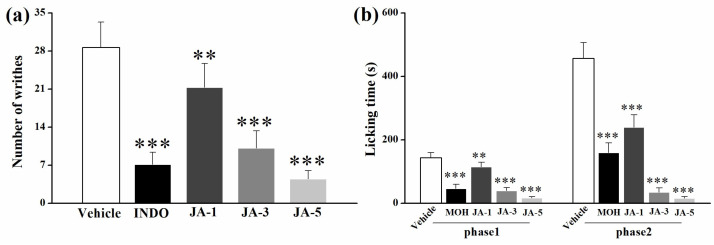
Antinociceptive effects of jegosaponin A (JA) in antinociceptive tests. JA at doses of 1, 3 and 5 mg/kg in the writhing test (**a**) and the formalin test (**b**). Vehicle: blank control, INDO: indomethacin, MOH: morphine hydrochloride. Data are displayed as mean ± SD. ** *p* < 0.01, *** *p* < 0.001 vs. vehicle group.

Since the fraction (EAE3-5) in front of JA at the dose of 10 mg/kg produced extremely significant antinociceptive responses (Figure 1), effects of different concentrations below 10 mg/kg (1, 3, 5 mg/kg) of JA were evaluated in antinociceptive tests. In the writhing test, compared with the control group, JA decreased the number of writhes significantly (*p* < 0.01) at the dose range of 1–5 mg/kg, and the antinociceptive effect displayed a dose-dependent manner (Figure 4a). The antinociceptive effect at 5 mg/kg was even stronger than that of the positive drug, indomethacin (INDO). In the formalin test (Figure 4b), JA exhibited a similar dose-dependent antinociceptive effect as reflected by the significantly (*p* < 0.01) reduced licking time in both phases in JA-treated mice.

### 2.4. Validation of Antinociceptive Mechanisms of Jegosaponin A

#### 2.4.1. Effects of Jegosaponin A on Anti-Inflammatory and Sedative Tests

**Figure 5:**
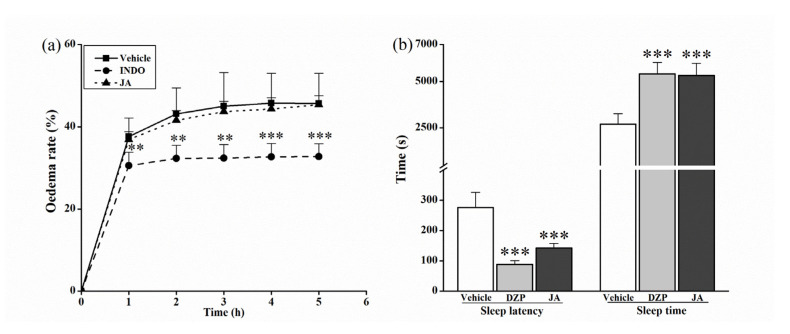
Effect of 3 mg/kg of jegosaponin A (JA) in the mouse paw edema test induced by carrageenan (**a**) and in the sleep test induced by the pentobarbital sodium (**b**). Vehicle: blank control, INDO: indomethacin, DZP: diazepam. Data are displayed as mean ± SD. ** *p* < 0.01, *** *p* < 0.001 vs. vehicle group.

In order to investigate the sedative effect and the anti-inflammatory activity of JA. the mouse paw edema test and the sleep test were performed. The results are shown in Figure 5. The reference drug INDO significantly (*p* < 0.01) blocked hind paw edema of mice within 5 h in the anti-inflammatory test. However, JA at the dose of 3 mg/kg showed no significant influence on paw edema when compared with the vehicle group. In the sleep test (Figure 5b), the reference drug diazepam (DZP) decreased the sleep latency and increased the sleep time significantly (*p* < 0.001). Similarly, for mice treated with JA at 3 mg/kg, the sleep latency was extremely (*p* < 0.001) shortened, and the sleeping time was extended considerably (*p* < 0.001).

#### 2.4.2. Effects of Calcium Ionophore and Antagonists on Antinociceptive Activities of JA

**Figure 6:**
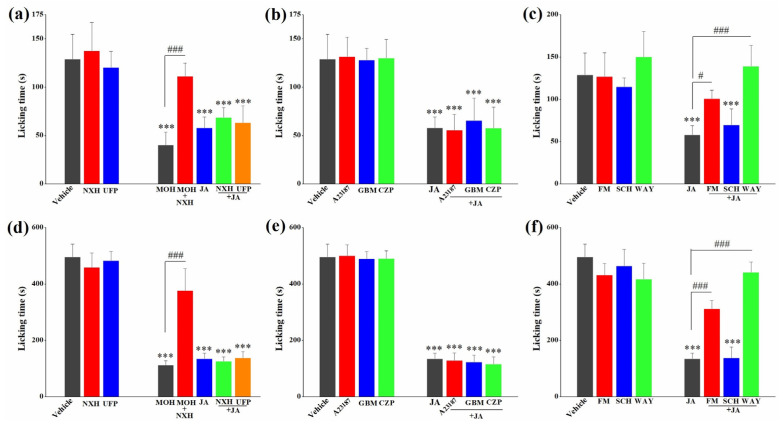
Effect of antagonists in the opioid system (**a**,**d**), antagonists and calcium ionophore in the ion channels system (**b**,**e**) and antagonists in the neurotransmitters system (**c**,**f**) on the antinociceptive activity of jegosaponin A (JA) in phase 1 (**a**–**c**) and phase 2 (**d**–**f**) of the formalin test. Vehicle: blank control, MOH: morphine hydrochloride. UFP: UFP-101, GBM: glibenclamide, CZP: capsazepine, FM: flumazenil, SCH: SCH23390 and WAY: WAY100635. Data are displayed as mean ± SD. *** *p* < 0.001 vs. vehicle group. # *p* < 0.05, ### *p* < 0.001 vs. model group.

Besides antagonists applied in our previous study, calcium ionophore A23187, GBM, CZP and UFP were added to investigate the antinociceptive pathway of JA. The results are shown in Figure 6. In both phases of the formalin test, pretreatment with A23187 or various antagonists showed no antinociceptive responses. In terms of the opioid system, the antinociceptive activity of morphine hydrochloride (MOH) was reversed completely (*p* < 0.001) by NXH in phase 1 and 2 of the formalin assay (Figure 6a,d). However, NXH and UFP failed to block the antinociceptive activity of JA in the animal model assays. Similarly, in regard to ion channels, the effect of JA was not inhibited by A23187, GBM or CZP anymore (Figure 6b,e). On the contrary, among antagonists of neurotransmitter receptors, FM and WAY affected the effect of JA evidently (Figure 6c,f). In particular, WAY completely abolished the antinociceptive response in both phases (*p* < 0.001).

#### 2.4.3. Effects of Jegosaponin A on Contents of Neurotransmitters in Brain Tissues

**Figure 7:**
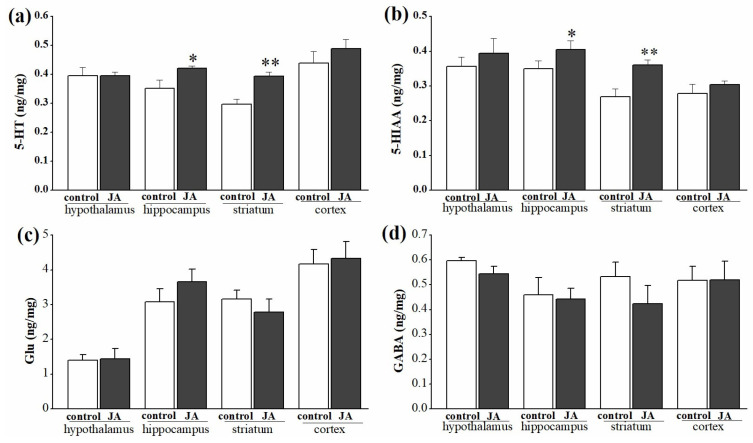
Effects of 3 mg/kg of jegosaponin A (JA) on the content of 5-HT (**a**), 5-HIAA (**b**), Glu (**c**) and GABA (**d**) in different brain regions of the mice. Data are displayed as mean ± SD. * *p* < 0.05, ** *p* < 0.01 vs. vehicle group.

Since the antinociceptive mechanism of JA was related to GABAergic and serotonergic systems according to the test of antagonists, contents of 5-HT, 5-HIAA, Glu and GABA were measured in four brain tissues. Compared with the control group, the content of the 5-HT and its metabolite (5-HIAA) in the JA group rose significantly (*p* < 0.05) in the hippocampus and (*p* < 0.01) striatum (Figure 7a,b). However, JA did not affect their contents in hypothalamus and cortex regions. By comparison, neither the content of Glu nor GABA in all four brain tissues were significantly changed by JA (Figure 7c,d).

### 2.5. Effects of Jegosaponin A on Open Filed and Elevated plus Maze Tests

It has been reported that hippocampal neurons are involved in the regulation of the emotional response to pain signals, and the striatum also plays a role in the regulation of emotional problems [20,21]. The increase of 5-HT content in these two tissues after JA treatment thus indicated that JA might affect the emotional problems caused by pain, such as anxiety. Behavioral tests (open field and elevated plus maze tests) for the anxiolytic evaluation of drugs were carried out.

**Figure 8:**
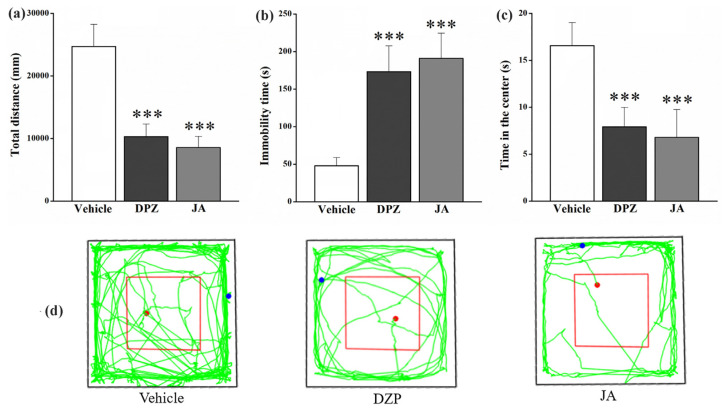
Effects of jegosaponin A (JA) at 3 mg/kg on the total distance (**a**), the immobility time (**b**) and the time in the center (**c**) in the open field test. Motion tracks of mice in vehicle, DZP and JA groups (**d**); red frame: center zone, red point: start, blue point: finish. Vehicle: blank control, DZP: diazepam. Data are displayed as mean ± SD. *** *p* < 0.001 vs. vehicle group.

In the open field test, DZP and JA extremely (*p* < 0.001) decreased the total distance (Figure 8a) and increased the immobility time of mice (Figure 8b), while times spent in the center zone by mice were shortened significantly (*p* < 0.001) (Figure 8c). Figure 8d provided motion tracks of mice in the vehicle, DZP and JA groups, and the decrease in locomotive activity was observed in both DZP and JA groups.

**Figure 9:**
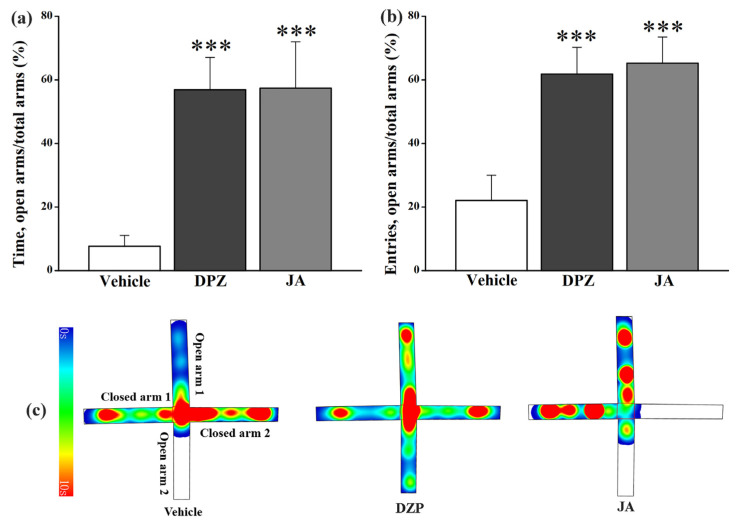
Effects of jegosaponin A (JA) at 3 mg/kg on the percentage of time in open arms (**a**) and the percentage of entries into open arms (**b**) in the elevated plus maze test. Heatmaps of mice in vehicle, DZP and JA groups (**c**). Vehicle: blank control, DZP: diazepam. Data are displayed as mean ± SD. *** *p* < 0.001 vs. vehicle group.

In the elevated plus maze test (Figure 9a,b), the same as DZP, the percentage of time in open arms and the percentage of entries into open arms increased tremendously (*p* < 0.001) for JA-treated mice. Heatmaps (Figure 9c) showed that DZP and JA increased the activity time of mice in open arms obviously.

## 3. Discussion

Flower extract and its active fraction (50 mg/kg) from *Styrax japonicus* were proven to have antinociceptive activity in our previous study, and the main compounds in the active fraction were glycosides, including kaempferol-3-*O*-rutinoside, forsythin and arctiin [7]. Kaempferol-3-*O*-rutinoside has been isolated from the safflower and reported to be able to relieve pain in writhing and formalin tests [22]. However, in this study, kaempferol-3-*O*-rutinoside at the dose of 50 mg/kg did not show a significant antinociceptive activity, neither did forsythin or arctiin (Figure S8). Finally, a saponin was determined to be the active compound through rigorous separation processes in combination with animal tests, and was then identified as JA. This was the first time that JA was isolated from the flower of *Styrax japonicus* and confirmed to be an antinociceptive compound. According to previous studies, JA was found firstly in the fruit of *Styrax japonicus* and was shown to have an anti-sweet action; it could also increase membrane permeability in prostate cancer cells and zebrafish embryos [18,19]. This study discovered a new bioactive property of JA, making its application in pharmacy possible.

In the mechanism study, JA did not effectively prevent paw edema caused by inflammation in mice (Figure 5a) but reduced the latency time to sleep and prolonged the sleeping time of mice in the pentobarbital sodium-induced sleep test, indicating the sedative effect (Figure 5b). Many compounds perform the antinociceptive effect depending on their anti-inflammatory activity [23,24] or the neurotransmitter system-mediated sedative effect [25]. Apparently, in the current study, the antinociceptive effect of JA was related to its sedative activity. Nonetheless, was the antinociceptive effect regulated by the neurotransmitter system?

Then, the antagonist for the GABA-A receptor (FM), the antagonist for the 5-HT_1A_ receptor (WAY) and the antagonist for the dopamine D1 receptor (SCH) were used to evaluate whether they could affect the antinociceptive effect of JA. Meanwhile, in order to avoid missing other possible antinociceptive mechanisms, calcium ionophore A23187, the ATP-sensitive K^+^ channel inhibitor (GBM) and the TRPV1 receptor antagonist (CZP), in regard to ion channels, and the non-selective antagonist at opioid receptors (NXH) and the selective antagonist at the NOP receptor (UFP), in regard to opioid systems, were added to investigate the antinociceptive pathways of JA. Calcium influx is important for evoking pain signal transmission; the activated TRPV1 receptor allows the passage of a variety of transmitters, such as substance P, somatostatin, neuronin A and kassinin, leading to the occurrence of pain. Additionally, the ATP-sensitive K^+^ channels are participants in peripheral pain [26,27,28]. Interestingly, only FM and WAY were the functional antagonists which attenuated the effect of JA to different degrees in both phases of the formalin test (Figure 6c,f). Furthermore, JA also increased contents of 5-HT and its metabolite (5-HIAA) significantly in the hippocampus and striatum (Figure 7). It was inferred that GABAergic and serotoninergic systems were involved in the antinociceptive mechanism of JA. It seemed that under the administration of JA, the hippocampus and striatum increased the secretion of 5-HT to inhibit the transmission of pain signals.

Neurons in the hippocampus are activated after pain signals transmit to the brain region, which promotes the formation of pain related to spatial memory and emotional responses [21]. The striatum is a complex functional body that coordinates the processing of movement, emotion and pressure [20]. Changes of the contents of 5-HT and the metabolite 5-HIAA in these brain tissues might indicate the influence of JA on the emotional aspect of pain. Anxiety is one kind of the most common emotions associated with pain, and it may be an essential part of the pain experience [29]. In this study, open field and elevated plus maze tests were applied to investigate the anxiolytic activity of JA. DZP and JA significantly restricted the locomotive activity of mice in the open field test (Figure 8), indicating that the antinociceptive effect, which was related to sedative activity, was mediated by the central nervous system [30]. However, both the reference drug and JA showed no anxiolytic activity since the time in the center zone decreased rather than increased significantly. This might be due to the low locomotive activity of mice in the DZP and JA treatment groups. On the contrary, DZP and JA significantly increased the percentage of time in open arms and the percentage of entries into open arms in the elevated plus maze test (Figure 9), indicative of the anxiolytic effect [31,32]. Therefore, JA might alleviate the anxiety caused by pain. However, more experimental studies are needed for further verification, such as establishing an anxiety model caused by pain to evaluate the anxiolytic effect of JA.

Finally, JA belongs to a pentacyclic triterpenoid saponin. The analysis of its antinociceptive pathways provides a reference for the study of the antinociceptive mechanism of triterpenoid saponins. Previous studies showed that some saponins might lead to hemolysis if under improper use, and the hemolytic activity was affected by the number of sugar units in the sugar chain [33,34]. Further toxicological tests on JA and its relevant secondary saponins need to be detailed in future.

## 4. Materials and Methods

### 4.1. Plant Materials

Flower materials were obtained from Nanjing, China in April 2021 and identified by Professor Yao Lei. The voucher specimen was stored at the herbarium of the Aromatic Plant Research Center of Shanghai Jiao tong University with a number (No. 20210503).

### 4.2. Drugs and Animals

Reference drugs in this study including MOH, INDO and DZP were donated by the Aromatic Plant Research Center. A23187, GBM, CZP and UFP were from Macklin Biochemical (Shanghai, China). NXH, FM, SCH, WAY, 5-HT, 5-HIAA, glutamate and GABA were from Sigma-Aldrich (St. Louis, MO, USA). D101 macroporous resin (particle size 16–60 mesh, moisture content 65–75%, surface area 500–550 m^2^/g) was from Huiying Instrument Business Department (Hangzhou, China).

Male ICR mice (25–30 g) were purchased from the SLAC Laboratory Animal (Shanghai, China) and kept in Laboratory Animal Center of Shanghai Jiao Tong University under a standard experimental environment. Animal assays were performed according to the regulation of Institutional Animal Care and Use Committee (IACUC) of Shanghai Jiao Tong University with a code (No. A2020010).

### 4.3. Isolation Procedure and Antinociceptive Tests

Flower materials (3.5 kg) were immersed in the ethanol solution (12 L) overnight, and the leach liquor was evaporated to yield 148.940 g paste. The paste was suspended in water 1000 mL and sequentially extracted with equal volumes of petroleum ether, ethyl acetate and n-butanol several times. The extractant was then evaporated to obtain 17.632 g PE, 66.564 g EAE and 12.176 g NBE together with 20.418 g AR. Each extract was subject to animal model tests; then EAE, the active extract, was allowed to pass through a column filled with D101 macroporous resin for fractionation. Thereafter, the column was eluted with ethanol and water with gradient concentrations (0%, 30%, 75% and 95%) to obtain EAE fractions (EAE1: 35.728 g, EAE2: 3.226 g, EAE3: 8.670 g and EAE4: 84 mg). Following the animal tests, EAE3 (2 g) as the active fraction was loaded to the silica gel (100–200 mesh) column chromatography and eluted with CH_2_Cl_2_–MeOH–H_2_O (30:1:0–15:15:4) gradient. Then, six fractions (EAE3-1: 659 mg, EAE3-2: 260 mg, EAE3-3: 559 mg, EAE3-4: 672 mg, EAE3-5: 1.786 g and EAE3-6: 377 mg) were obtained, and EAE3-5 (1 g), the active fraction, was repeatedly loaded into the semi-preparative HPLC system coupled with a C18 OBDTM Prep Column (5 μm, 10 mm × 150 mm) for further fractionation. The mobile phase consisted of (A) water with 0.1% formic acid and (B) acetonitrile. A gradient elution process was performed, and two compounds were eluted out of the column, which were named Compound **1** (182 mg) and Compound **2** (87 mg). Compound **1** was ascertained to be the active substance through pre-screening. The purity of the Compound **1** was 98.5% through the ^1^H-QNMR determination (Supplementary Materials) [35].

Fractions were assessed by writhing and formalin tests. In each test, mice were pre-administered with 10 mL/kg of blank control (physiological saline), 10 or 50 mg/kg of fractions and 10 mg/kg of reference drug (MOH or INDO). Then, animal models were built with six replicates for each group. For writhing test, animals were intraperitoneally treated with 10 mL/kg of acetic acid solution (0.8%, mL/mL) after 30 min of drug treatment. The number of writhing movements of mice during 30 min was counted. In the formalin test, 30 min after drug treatment, 30 μL of formalin solution (1%, mL/mL) was subcutaneously injected into the right-hind paw of mice. The paw licking time was measured during the 10 min (Phase 1) and the 10–30 min (Phase 2) periods.

### 4.4. Identification of the Active Compound ***1***

#### 4.4.1. UHPLC-QE-MS Analysis

The UHPLC-QE-MS system (Vanquish, Thermo Fisher Scientific, Waltham, MA, USA) equipped with a C18 column (100 × 2.1 mm, 1.7 μm) and an ESI source was used to analyze the active Compound **1**. The mobile phase consisted of water with 0.1% formic acid (A) and acetonitrile with 0.1% formic acid (B). A gradient elution program was as follows: 0–10 min, from 95% to 0% A; 11–12 min, 100% B; and 12–14 min, 95% A. The injection volume was 5 μL and the column flow rate was 0.4 mL/min. Data were acquired in full MS and product ion MS/MS scan in ESI positive and negative modes over a *m*/*z* range of 50–2000. The normalized collision energy was performed to form fragment ions, and nitrogen was employed as dissociation gas. Spray voltage was 3.2 kV (positive) or −3.0 kV (negative). Data were obtained by the Xcalibur 3.0. software, and the functional groups of the molecular structure were inferred according to the MS/MS spectra.

#### 4.4.2. Determination of the Absolute Configuration of Sugars

Acid hydrolysis of Compound **1** and analysis of absolute configuration of its sugar groups were performed referring to the previous study with partial modification [36]. Specifically, Compound **1** (2 mg) in 2 M TFA (2 mL) was stirred at 100 °C for 4 h. After cooling, ethyl acetate (2 mL) was added to the aqueous solution three times. Each time the mixture was vortexed thoroughly and the organic phase containing organic impurities was discarded. The aqueous phase containing the target actives was collected and evaporated under the reduced pressure. The residue along with L-cysteine methyl ester hydrochloride (2 mg) were dissolved in 1 mL anhydrous pyridine and warmed at 60 °C for 2 h. Then the reagent trimethylsilylimidazole (0.2 mL) was added to the mixture, followed by warming at 70 °C for 40 min. After evaporating the solution, the residue was re-dissolved in water (1 mL) and extracted by hexane (1 mL) for GC-MS analysis.

Agilent GC-MS (7890B-5977) equipped with a DB-WAX capillary column (30 m × 250 μm, 0.25 μm film thickness) was applied to analyze the solution. A 2 μL solution was injected into the system for analysis with a split ratio of 10:1. The injector temperature was 260 °C, and the column flow rate was 1 mL/min. The oven temperature was programmed at 50 °C for 3 min, then increased to 220 °C at a rate of 5 °C/min, keeping for 20 min.

#### 4.4.3. IR and NMR Analysis

The mid infrared spectral information of Compound **1** was collected by a microscopic imaging infrared spectrometer (iN10 MX, Thermo, USA) with the spectral range of 4000-450 cm^−1^. The spectral data of ^1^H-NMR, ^13^C-NMR, ^1^H-^1^H shift correlation spectroscopy (COSY), heteronuclear single quantum coherence (HSQC) and heteronuclear multiple bond connectivity (HMBC) were obtained by a 700MHz nuclear magnetic resonance spectrometer (AVANCE NEO, Bruker, Billerica, MA, USA) with the scanning range ^1^H: −1–13 ppm, ^13^C: −12–230 ppm.

### 4.5. Antinociceptive Effects of Jegosaponin A

Antinociceptive effects of JA at doses of 1, 3 and 5 mg/kg were evaluated in writhing and formalin assays. The procedure was the same as described above. Intraperitoneal injection was selected as the route of administration consistent with the previous study [7] and based on its own advantages. It is shown that drugs injected intraperitoneally diffuse into the visceral peritoneum through the capillaries in the spleen and mesentery or are absorbed through the lymph, with a large absorption area, fast effect and low invasion, which could avoid potential degradation [37].

### 4.6. Analysis of Antinociceptive Mechanisms of Jegosaponin A

#### 4.6.1. Mouse Hind Paw Edema and Sleep Tests

Carrageenan-induced mouse hind paw edema and pentobarbital sodium-induced sleep tests were employed to reveal whether the antinociceptive effect of JA is related to its anti-inflammatory capacity or sedative activity. In the hind paw edema test, blank control, 3 mg/kg of JA or 10 mg/kg of the reference drug (INDO) were injected into the mice of each group intraperitoneally. After 30 min of drug administration, 30 μL of carrageenan (1%, *v*/*v*) was injected into the right hind paw of mouse individually. Then the paw edema of each mouse was detected at the following time points (0, 1, 2, 3, 4, 5 h).

In the pentobarbital sodium-induced sleep test, blank control, DZP (2 mg/kg) or JA were administrated to animals. After 15 min of drug administration, 50 mg/kg of sodium pentobarbital was injected into each mouse intraperitoneally. The sleep latency and sleep time of animals were recorded.

#### 4.6.2. Antagonists and Calcium Ionophore Tests of Jegosaponin A

To reveal the antinociceptive mechanism of JA, apart from the opioid system and neurotransmitter systems as investigated in our previous study, calcium ion influx, ATP-sensitive K^+^ channels and TRPV1 were also investigated. Mice were pretreated with calcium ionophore A23187 (1 μg/kg), GBM (10 mg/kg), CZP (5 mg/kg), NXH (7.5 mg/kg), UFP (10 nmol/mouse), FM (10 mg/kg), SCH (0.05 mg/kg) or WAY (0.7 mg/kg). After 15 min, vehicle, reference drug (MOH) or JA (3 mg/kg) was administrated to mice. After 30 min, the formalin test was performed.

#### 4.6.3. Evaluation of 5-HT and 5-HIAA, Glu and GABA in Brain Tissues of Mice

After the formalin test, mice were immediately euthanized, and relevant brain tissues were dissected individually from the mice brains. Neurotransmitters and metabolites were extracted from different tissues as described in the literature [38,39]. The determination was performed using the ultraperformance liquid chromatography with a fluorescence detector (UPLC-FLD) equipped with a C18 column (100 × 2.1 mm, 1.7 μm, Waters). The composition of the mobile phase was 0.1% formic acid in water (A) and 0.1% formic acid in acetonitrile (B). Both 5-HT and 5-HIAA were detected using isocratic elution (95% A and 5%B), while a gradient elution program was applied to Glu and GABA as follows: 0–8 min, 95% (A); 8–10 min, 10% (A); and 10–13 min, 95% (A).

#### 4.6.4. Anxiolytic Test of Jegosaponin A

Open field and elevated plus maze tests were used to evaluate the anxiolytic activity of drugs [40,41]. As for the open field test, the apparatus consisted of a black square area (45 × 45 cm) and plastic walls (40 cm) around. Mice were injected with blank control (10 mL/kg), DZP (2 mg/kg) or JA (3 mg/kg). After 15 min of drugs administration, animals were arranged in the center of the box individually and the total distance, immobility time, as well as the time in the center zone of animals, were recorded during 5 min.

In the elevated plus maze test, the maze consisted of four arms (two open arms: 30 × 6 cm and two closed arms: 30 × 6 × 15 cm) and a cross-like area (6 × 6 cm platform in the middle) [42]. Then, 15 min after drug administration, animals were individually arranged on the cross-like area with head pointing toward open arms. The total time that mouse spent in open arms or closed arms and numbers of entries into two arms were recorded during the 5 min test period. Percentages of time in open arms and percentages of entries into open arms were also calculated.

### 4.7. Statistical Analysis

The data from animal tests were analyzed using one-way ANOVA coupled with Tukey’s post-hoc test. Two-tailed Student’s t-test was used to analyze the data when there were two groups. All data were processed by RStudio-1.1.456.

## 5. Conclusions

In conclusion, there are very few reports on the medicinal use of compounds from the flower to the best of our knowledge. It was the first time that JA was isolated from the flower of *Styrax japonicus* and was demonstrated to possess significant antinociceptive effects in writhing and formalin tests. This study provided hints for its potential application in pharmacy. In the mechanism study, JA could not effectively inhibit the inflammation process of mice in the paw edema test but displayed a significant sedative effect in the sleep test. This indicated that the antinociceptive effect of JA was related to the neurotransmitter system, with GABAergic and serotonergic systems involved, to be specific, according to the test of antagonists. In addition, since JA showed an anxiolytic activity in the elevated plus maze test, it might affect the emotional aspect of pain. In future, the effect of JA on anxiety caused by pain needs to be investigated in depth.

## Figures and Tables

**Table 1:** ^1^H (700 MHz) and ^13^C (176 MHz) NMR spectroscopic data for compound **1** in pyridine-d5.

Position		δ_C_	δ_H_ (*J* in Hz)
Aglycon	1	38.88	0.794 ^o^, 1.330 ^o^
	2	26.60	1.658 m, 2.066 m
	3	89.77	3.204 dd (11.76, 4.2)
	4	39.84	
	5	55.85	0.733 t (12.81)
	6	18.61	1.495 (2H) m
	7	33.29	1.295 m, 1.562 m
	8	40.18	
	9	47.03	1.677 m
	10	36.90	
	11	23.98	1.754 m,1.839 ^o^
	12	123.97	5.384 m
	13	143.02	
	14	41.85	
	15	34.84	
	16	68.22	4.439 ^o^
	17	48.15	
	18	40.24	3.073 ^o^
	19	47.37	1.391 m, 3.074 m
	20	36.64	
	21	79.56	6.602 dd (10.15, 1.96)
	22	74.48	6.267 d (9.30)
	23	28.12	1.175 s
	24	16.90	1.061 s
	25	15.78	0.786 s
	26	17.02	1.175 s
	27	27.61	1.834 s
	28	63.88	3.384 d (10.43), 3.623 dd (10.57, 3.22)
	29	29.70	1.098 s
	30	20.31	1.328 s
AC	1	171.16	
	2	21.05	1.895 s
Tig	1	168.19	
	2	129.66	
	3	137.02	7.108 q (6.54)
	4	14.39	1.643 d (7.00)
	5	12.80	1.957 s
GlcA	1	105.58	4.89 d (5.88)
	2	79.87	4.747 m
	3	82.89	4.757 m
	4	71.25	4.486 m
	5	77.53	4.584 m
	6	172.38	
Glc	1	102.90	5.939 d (7.42)
	2	76.50	4.159 t (8.12)
	3	78.36	4.442 m
	4	72.75	4.808 brs
	5	77.21	4.27 m
	6	63.73	4.352 m, 4.701 m
Gal	1	101.60	6.199 d (7.70)
	2	76.63	4.737 m
	3	76.23	4.501 m
	4	71.33	4.488 m
	5	77.10	4.489 m
	6	62.03	4.319 m, 4.363 m
Rha	1	102.57	6.256 ^o^
	2	74.24	4.392 m
	3	72.81	4.713 m
	4	74.07	4.225 m
	5	69.96	4.903 m
	6	18.48	1.437 d (6.09)

^o^ Overlapped with other signals.

## Data Availability

Data are contained within the article.

## Supplementary Materials

The following supporting information can be downloaded at: https://www.mdpi.com/article/10.3390/molecules28052243/s1. Figure S1: Antinociceptive effects of fractions in antinociceptive tests, effects of 50 mg/kg of fractions isolated from the ethanol extract (a,b), 10 mg/kg of fractions from EAE (c,d); Figure S2: MS/MS spectrum in positive mode of compound **1**; Figure S3: (a) ^1^H (700 MHz) and (b) ^13^C (176 MHz) NMR spectrums of compound **1**; Figure S4: ^1^H-^1^H shift correlation spectroscopy of compound **1**; Figure S5: Heteronuclear single quantum coherence spectroscopy of compound **1**; Figure S6: Heteronuclear multiple bond connectivity spectroscopy of compound **1**; Figure S7: IR spectral information of compound **1**; Figure S8: Effects of kaempferol-3-O-rutinoside, forsythin and arctiin in writhing (a) and formalin tests (b); Figure S9: The spectrum of the ^1^H-QNMR of JA and hydroquinone.

## Sample Availability

Samples of the compounds are not available from the authors.
