# Supplementary material for: Involvement of GABAergic and Serotonergic Systems in the Antinociceptive Effect of Jegosaponin A Isolated from Styrax japonicus
Source: Molecules. 2023 Feb 28;28(5):2243. doi: 10.3390/molecules28052243 (PMC10005120; doi:10.3390/molecules28052243)
Supplement: Supplementary file 1 [file molecules-28-02243-s001.zip › Supplementary Data.pdf]

Supplementary Data

S1. Antinociceptive effects of sub-extracts

Antinociceptive effects of petroleum ether extract (PE), ethyl acetate extract (EAE), n-butanol extract (NBE) and aqueous residues (AR) from the ethanol extract and the fraction EAE are shown in Figure S1.

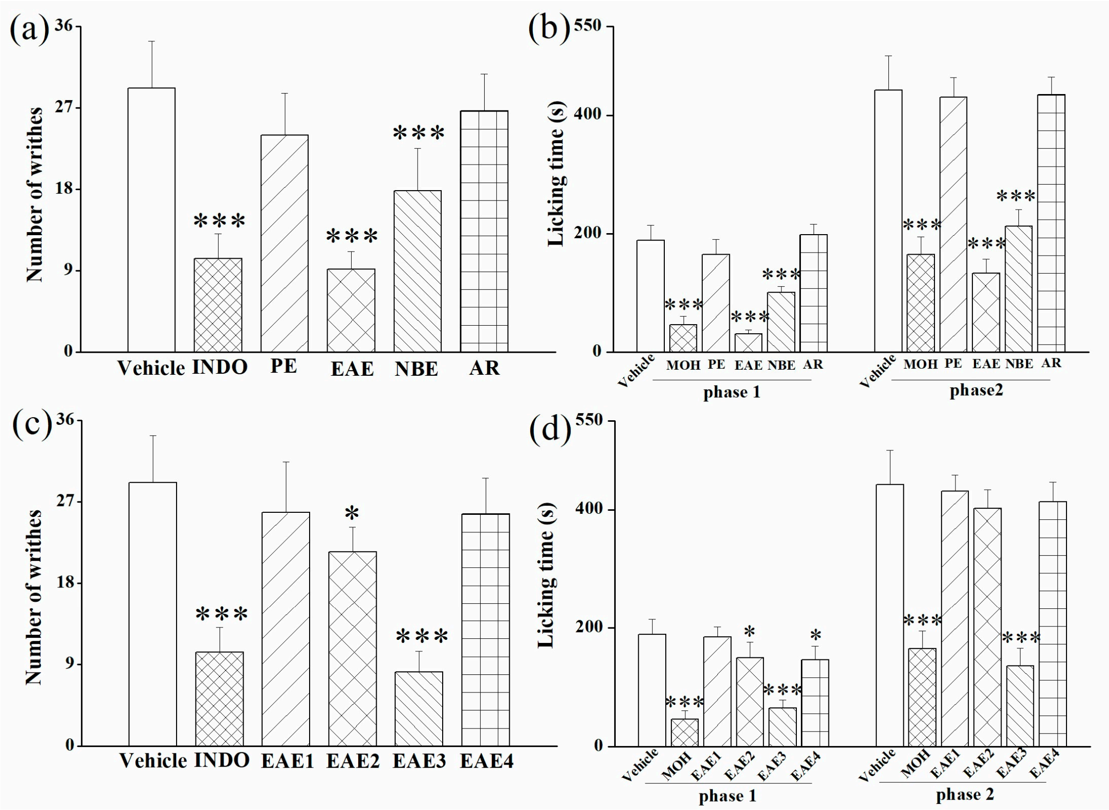

Figure S1. Antinociceptive effects of fractions in antinociceptive tests, effects of 50 mg/kg of fractions isolated from the ethanol extract (a, b), 10 mg/kg of fractions from EAE (c, d). Data are displayed as mean ± SD. $*p < 0.05$, $***p < 0.001$ vs. vehicle group.

S2. Fragment ions of compound 1

MS/MS spectrum in positive mode of compound 1 was shown in Figure S2. Seven major fragments were detected. Fragment 1 showed a ion $[M+H]^+$ at m/z 1261.6243, fragment 2 ion $[M-18+H]^+$ at m/z 1243.6108, fragment 3 ion $[M-18-146+H]^+$ at m/z 1097.5524, fragment 4 ion $[M-18-146-162+H]^+$ at m/z 935.4998, fragment 5 ion $[M-18-146-162-162+H]^+$ at m/z 773.4464, fragment 6 ion $[M-18-146-162-162-176+H]^+$ at m/z 597.4148, fragment 7 ion $[M-18-146-162-162-176-18+H]^+$ at m/z 579.4045. Which indicated that the molecule might contain two hydroxyl, one methylpentose, two hexoses and one hexuronic acid groups.

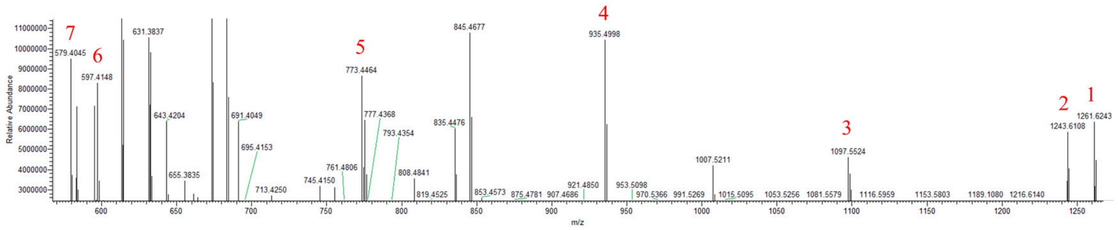

Figure S2. MS/MS spectrum in positive mode of compound 1.

S3. NMR spectrums of compound 1

^{1}H-^{1}H shift correlation spectroscopy (COSY), heteronuclear single quantum coherence (HSQC) and heteronuclear multiple bond connectivity (HMBC) information were obtained. Spectrums were presented in Figure S3-6.

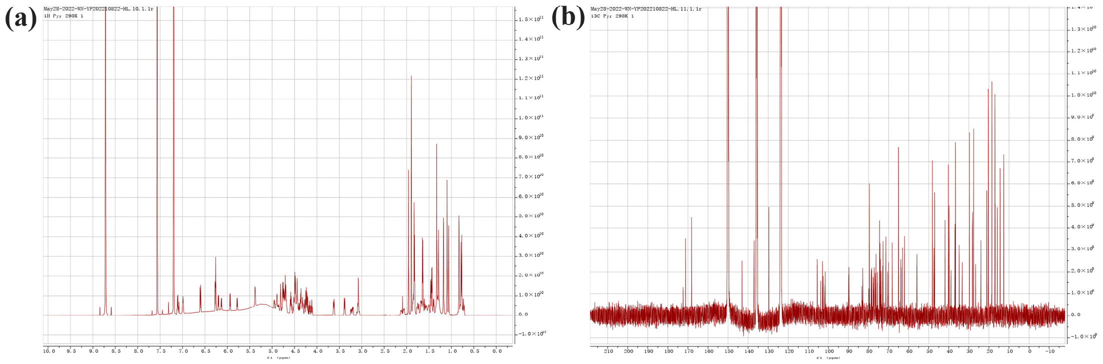

Figure S3. (a) ^{1}H (700 MHz) and (b) ^{13}C (176 MHz) NMR spectrums of compound 1.

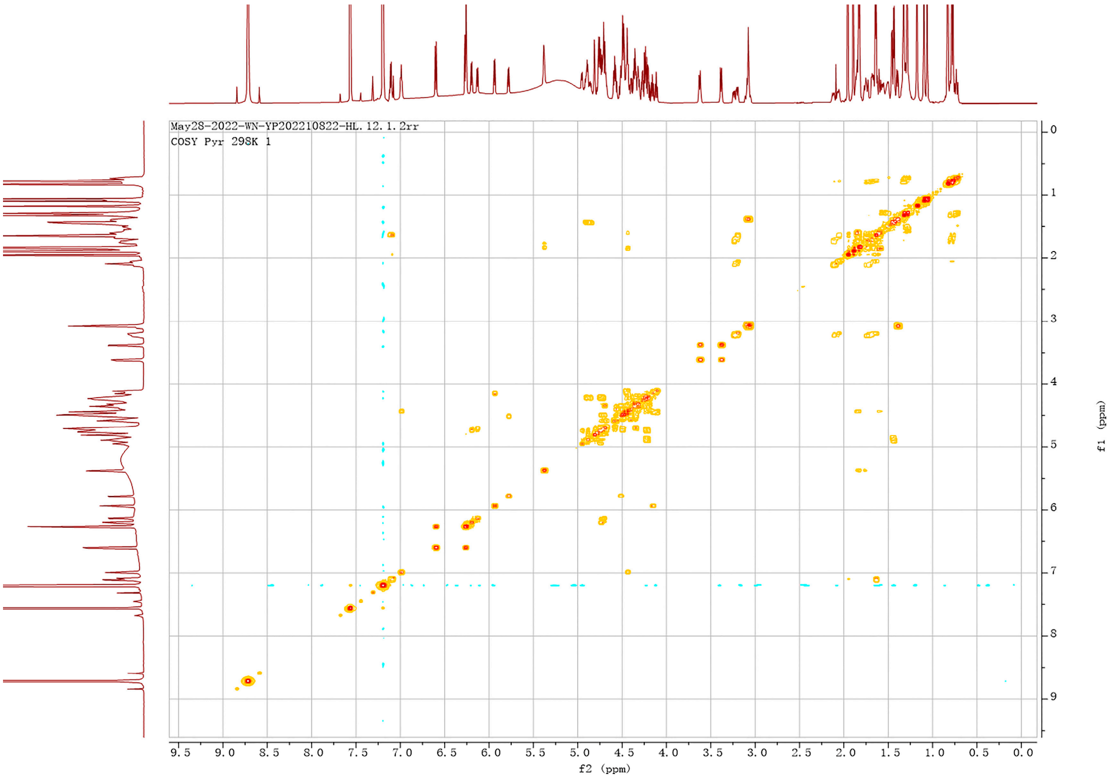

Figure S4. ^{1}H-^{1}H shift correlation spectroscopy of compound 1.

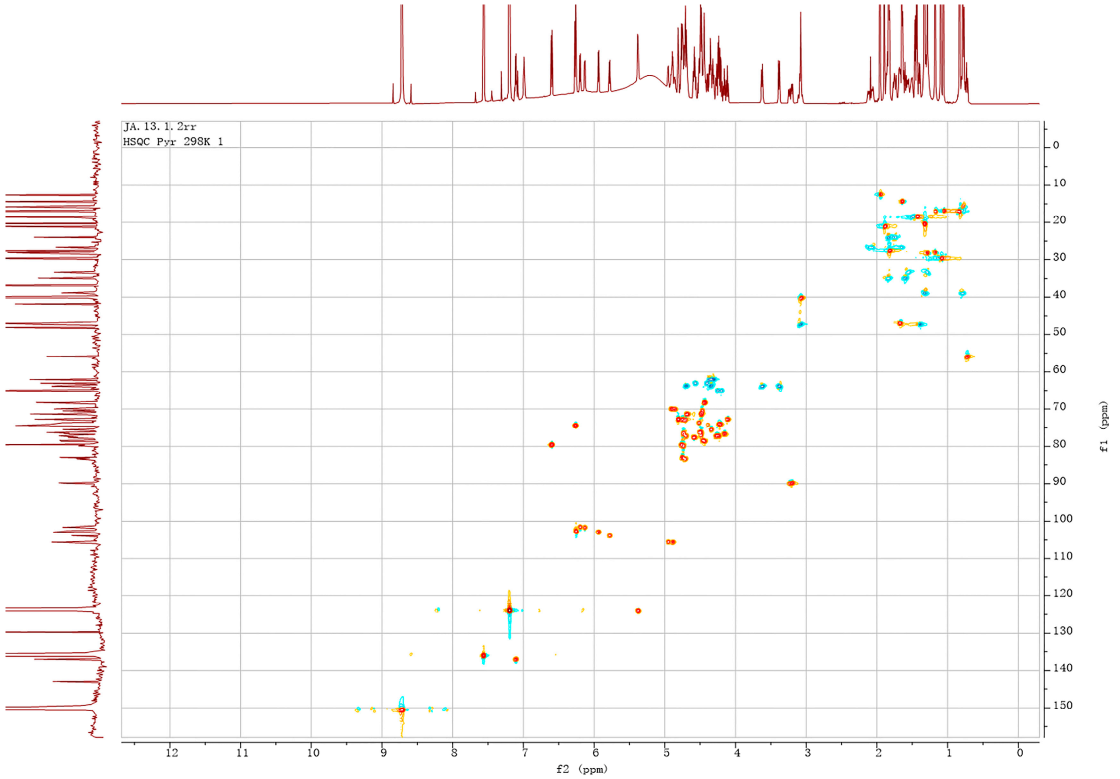

Figure S5. Heteronuclear single quantum coherence spectroscopy of compound 1.

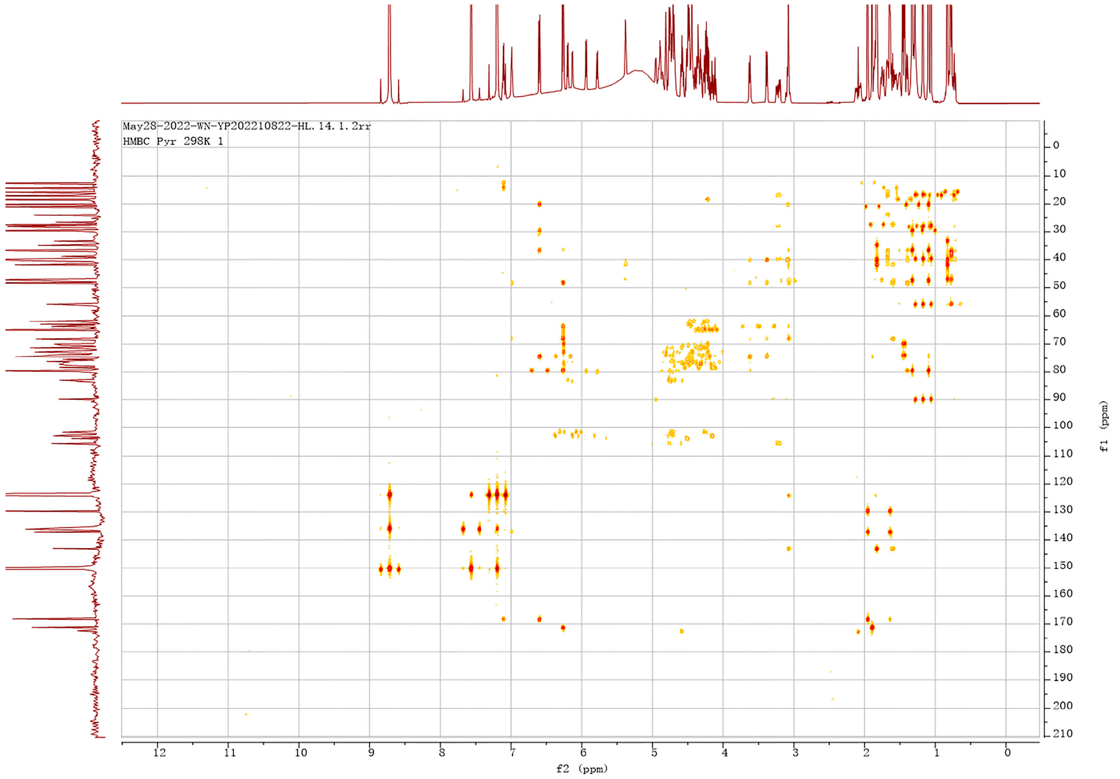

Figure S6. Heteronuclear multiple bond connectivity spectroscopy of compound 1.

S4. Infrared spectrum of compound 1

The mid infrared spectral information of compound 1 was shown in Figure S7. Signals of hydroxyl and several carbonyl moieties were detected.

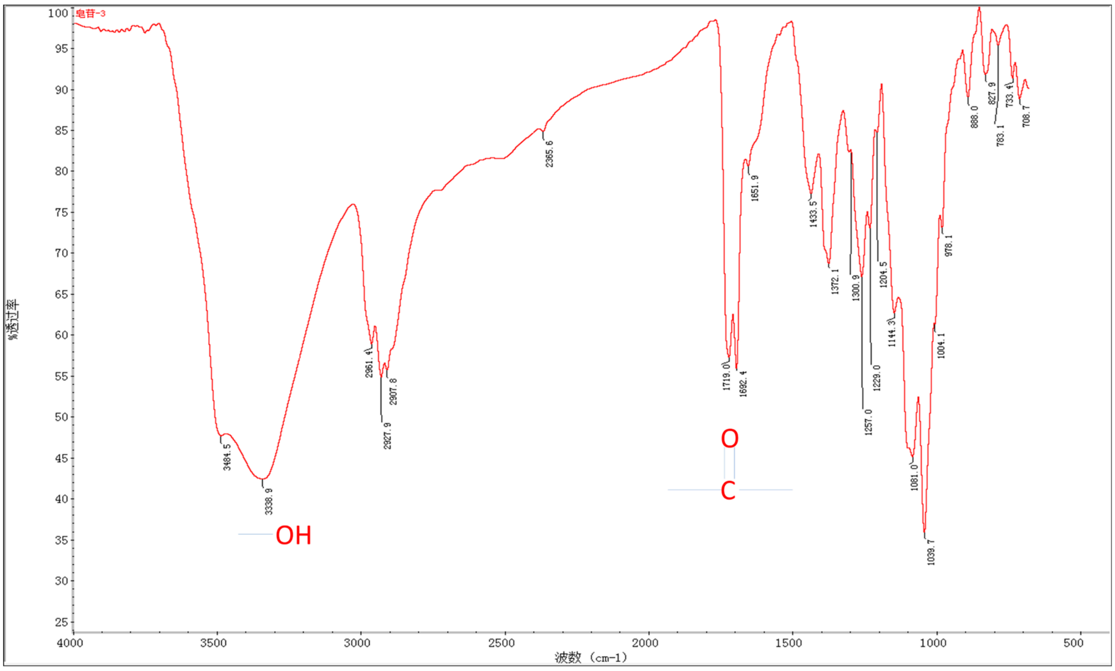

Figure S7. IR spectral information of compound 1.

S5. Analgesic effects of kaempferol-3-O-rutinoside, forsythin and arctiin

Effects of kaempferol-3-O-rutinoside, forsythin and arctiin were evaluated in writhing and formalin tests. kaempferol-3-O-rutinoside at the dose of 50 mg/kg as same as the active fraction of the flower extract in which enough to trigger the analgesic response did not show a significant analgesic activity, neither did forsythin and arctiin (Figure S8).

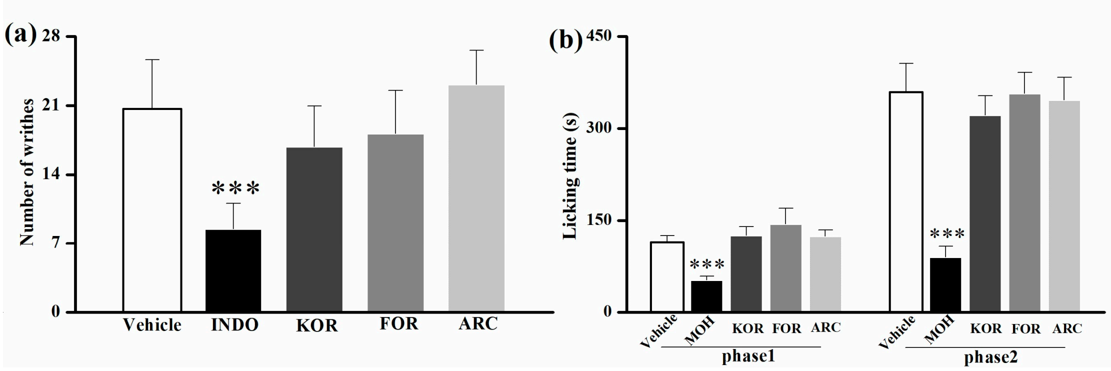

Figure S8. Effects of kaempferol-3-O-rutinoside, forsythin and arctiin in writhing (a) and formalin tests (b). INDO (indomethacin, 10 mg/kg), MOH (Morphine hydrochloride, 10 mg/kg), KOR (kaempferol-3-O-rutinoside, 50 mg/kg), FOR (forsythin, 50 mg/kg), ARC (arctiin, 50 mg/kg).

S6. The purity determination of JA

The purity determination of JA (Compound 1) reference to the ^{1}H-QNMR method [35]. The purity of main component X can be calculated directly from the NMR spectrum using the following formula:

$$P_x = \frac{I_x}{I_{std}} \times \frac{N_{std}}{N_x} \times \frac{M_x}{M_{std}} \times \frac{W_{std}}{W_x} \times P_{std}$$

where, I, N, M, W and P are integral area, number of nuclei, molar mass, gravimetric weight and purity of analyte (X) and standard (STD), respectively.

In this study, the reference standard was hydroquinone. The spectrum of the ^{1}H-QNMR was shown in Figure S9. I_{std} = 31.94, N_{std} = 6, M_{std} = 110.11 g/mol, W_{std} = 2.021 g, and P_{std} = 99.5%; I_{JA} = 4.96, N_{JA} = 5, M_{JA} = 1261.4 g/mol, W_{JA} = 4.357 g.

$$P_{JA} = \frac{4.96}{31.94} \times \frac{6}{5} \times \frac{1261.4}{110.11} \times \frac{2.021}{4.357} \times 99.5 = 98.5$$

The purity of JA was 98.5%.

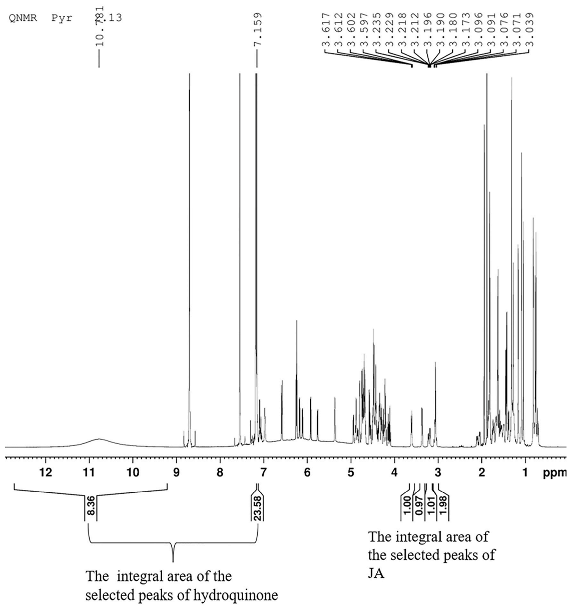

Figure S9. The spectrum of the ^{1}H-QNMR of JA and hydroquinone

Abbreviations

JA, jegosaponin A; FM, flumazenil; WAY, WAY100635; UHPLC-QE-MS, ultrahigh-performance liquid chromatography Q extractive mass spectrometry; IR, Infrared; NMR, nuclear magnetic resonance; GBM, glibenclamide; CZP, capsazepine; TRPV1, Transient receptor potential vanilloid type1; NXH, naloxone hydrochloride; UFP, UFP-101; SCH, SCH23390; PE, petroleum ether extract; EAE, ethyl acetate extract; NBE, n-butanol extract; AR, aqueous residues; INDO, Indomethacin; DZP, Diazepam; MOH, Morphine hydrochloride; UPLC-FLD, ultraperformance liquid chromatography with a fluorescence detector.
